# Cautions Respecting the Inoculation of Cow-Pox

**Published:** 1801-01

**Authors:** Jonathan Stokes

**Affiliations:** Chesterfield


### *Cautions respecting the Inoculation of Cow-Pox, by* Dr. Stokes.

### To Dr. BRADLEY.

Dear Sir,

THE discovery of a disease in a quadruped of a genus so totally distinct from man, which, without exciting any considerable disturbance in the constitution, renders him insensible to the action of the most generally fatal malady to which he is subject, is a phænomenon which has arrested the attention of every enlightened physiologist; and the reception which this discovery has met with, deserves the attention of the metaphysician. The common people, as far as I have observed, receive it with gladness, as it comes to them free of expence, and some of them have been heard to speak of it as a blessing sent from God. They do not express any more repugnance to the insertion of pellucid lymph from the arm of a neighbour's child into that of their own offspring, because originally taken from a vesicle on the nipple of a cow, than they do to feed them with the milk or flesh of that animal. Their minds are no longer held in suspence, whether they shall consent to give their child a malady which may terminate in its death, with a view to preserve it from a disease which they sometimes observe to be a very slight one. The inoculator can now assure them, that their child *will not die of inoculation*, and every new inoculation confirms to them the truth of his assertion. The middling classes, who are in the habit of being guided by the practitioners they employ, take their opinion on the subject, and sometimes commission them to inoculate their children "with either kind of pock." The upper classes, who read and see a variety of practitioners, judge for themselves; and those among them who are philanthropists and converts to the new faith, inoculate their own children and those of the poor together. Mr. Fermor's pamphlet will, I doubt not, make many converts among this class, and I hope his example will have many followers. With regard to our medical brethren, the metaphysicians will tell us, that we are not in general to look up for support from the timidity of advancing years, and they will forbid us to be sanguine in promising ourselves the sanction of those who are past 70. I have, however, the pleasure to observe, that Dr. Hunter, of York, in a speech delivered before the Directors of the Dispensary in that city, recommends a general vaccine inoculation, with all the a-dor and enthufiafm of youth. Other veterans in medicine will, I doubt not, ftand forward in the fame caufe; and fhould we live to fo advanced a period of life, may we prove equally alive to the benefits likely to be derived from fome other difcovery equally beneficial to mankind! But the moft formidable clafs of opponents which the new inoculation has to encounter, are thofe who calculate their future income by the quantum of expected difeafe; yet thefe we might foon make converts to the new doctrine, if we could convince them that the rapid increafe of population would amply compenfate for the apprehended defalcation of their accuftomed profits. But the patrons of the new inoculation need to fear no oppofition, if in fteadily purfuing the practice they ftudy Nature, and guarding themfelves againft the feduction of falfe theory, hold themfelves ever ready publicly to acknowledge any errors into which they may have been led by inaccurate or limited obfervation. They ought to keep up a conftant fucceffion of inoculations, fo that recent matter may be always in readinefs; but if the Small-pox fhould break out in any family, all thofe who have not had the difeafe fhould be inftantly inoculated with Cow-pox virus, even though labouring under other difeafes; the parents being at the fame time reminded, that the Cow-pox is no prefervative againft fuch difeafes, nor even againft Small-pox, provided the fubject of inoculation has been expofed to its miafma. In fuch cafes the inoculator fhould make feveral punctures, and the patient, if convenient, fhould be inftantly removed to a fituation where they may not be expofed to the contagion of Small-pox: Additional fecurity may be obtained by making a frefh inoculation every day till marks of infection appear. Some of the beft writers on the fubject, fpeak of the difficulty of communicating the difeafe, and feem to think it a virus lefs active than that of Small-pox. My experience has led me to draw an oppofite conclufion. My method is to introduce the lancet into the Cow-pox veficle. In a few moments after withdrawing it, a fmall pellucid globule appears on the cuticle, which I fcrape off by the edge of the lancet; I then turn the lancet, and fcrape the cuticle ftill wet with the other edge; I then introduce the lancet between the cuticle and cutis of the left arm of the patient who is to be inoculated, holding the fide of the lancet parallel to the arm, the arm hanging in its natural pofition, but the point of the lancet perpendicular to the horizon, that the virus may flow downwards into the minute wound, and its introduction I endeavour to facilitate by gently moving the lancet upwards and downwards. I fometimes turn the lancet and introduce it into the fame punctures, which are generally vifible, in children at leaft, whofe cuticle is fo thin, that that it is difficult to introduce the lancet without fome flight effufion of blood. Thefe precautions are peculiarly neceffary where the Small-pox has already made its appearance in the family. In two inftances where the patients were inoculated with recent virus in the afternoon of the day on which the Small-pox broke out in another child in the family, no puftules appeared excepting thofe on the arm; but in Job Webfter, two months old, inoculated in both arms with virus taken on a lancet and carried a quarter of a mile, puftules appeared on the 4th day on the lower limbs, at the fame time that one of the punctures became elevated into a red pimple, which I faw on the 4th, but it was not till the 6th that I faw the puftules: the inoculated puftule was now become orbicular, with a flight depreffion in the middle. On the lower limbs and the lower part of the trunk we counted fixteen puftules. The largeft, which was on the right knee, was orbicular, and depreffed, exactly as that on the arm, but rather larger, and furrounded by an efflorefcence of very minute papillæ, fimilar to what I have fometimes obferved round puftules on the arms of Cow-pox patients. The child's mother, one of my female pupils, and Mr. Walker, furgeon, who is in the habit of inoculating with both Small-pox and Cow-pox, were of opinion with me, that it was a Cow-pox puftule, and I became for a time a convert to the opinion of Dr. Woodville, that thefe puftules were the confequence of the action of a variolated atmofphere. On the 7th, the puftule on the arm meafured in diameter $1\frac{1}{2}$ tenth of an inch, and was furrounded by a very flight rednefs; that on the knee was $2\frac{1}{4}$ tenth of an inch, and was of a dirty brownifh hue in the middle. There was a lenticular puftule on the left temple, $1\frac{1}{4}$ tenth of an inch in diameter, and two or three on the lower part of the abdomen, which were of a whitifh hue in the middle. The reft on the body refembled that on the knee, excepting two on the middle of the abdomen, which appeared like variolous puftules. The lower limbs and the inferior part of the trunk were of a redder hue than the reft of the body, fimilar to the appearance of the fkin in fcarlatina. On the eighth day, the puftule on the arm was increafed to two-tenths of an inch, and the furrounding fkin was more inflamed. The puftule on the knee was browner in the middle, and the furrounding minute puftules larger; and more puftules made their appearance on the upper part of the body. The point of a lancet was paffed into the margin of the puftule on the knee, and into fome of the furrounding puftules; but neither lymph nor pus exuded. The lancet was barely moiftened, and being carried to the diftance of about a hundred yards, was inferted into the arm of a child four years old. Another lancet was introduced into the puftule on the arm, but neither lymph nor pus exuded. The moiftened lancet was carried to the fame diftance, and inferted into the arm of another child, 1 ½ year old. On the ninth, the puftule on the arm was furrounded by an inflamed areola of about an inch in diameter; that on the knee was larger, as alfo its furrounding minute puftules, and more puftules had appeared on the upper part of the body, and one or two on the face. On the tenth, the puftule on the arm was larger, but the inflammation round it as the day before; thofe on the limbs were rather larger, and more had appeared on the face. On the eleventh, the puftule on the arm meafured 3 ½ tenths of an inch, and was of a whitifh afh colour, and the areola was become rather paler; thofe on the knee were covered with a yellow cruft.

Elizabeth Titley, the child *inoculated from the pustule on the arm* of Job Webfter, went through the Cow-pox in the ufual way. I was informed that on the feventh day the punctures inflamed, and I examined the arm on the eleventh; the puftule meafured 3 ½ tenths of an inch, and the areola 1 inch eight-tenths in diameter. On the twelfth, the greater part of the areola had nearly difappeared, but it had extended with an irregular outline towards the axilla. The puftule afforded pellucid lymph.

In Hannah Titley, the child *inoculated from the pustules on the knee* of Job Webfter, I was informed the punctures became inflamed on the ninth, two days after her fifter's. On the eleventh, when I firft faw the child, the puftule on the arm meafured 2 ¼ tenths of an inch, was depreffed in the middle, and was furrounded by an inflamed fpace half an inch diameter, of a deep red. Her mother obferved, that fhe had been hot and not well for two or three days, and that fhe had eaten very little. In the evening a puftule appeared on her right arm. On the twelfth, the puftule on her right arm appeared elevated, with a flight depreffion at the top, and others had appeared on all parts of her body. She took little or no food, and her pulfe was 120. On the thirteenth, the puftule from inoculation appeared irregular, with an angular margin, with feveral fmall ones round it, and more puftules had come out. On the fixteenth, the puftule being opened by a needle, a pellucid lymph exuded. The puftule and inflammation round it meafured 1 inch 1-tenth in length. On the feventeenth, the puftule was fpread wider and longer, with an irregular margin, exactly like that of inoculated Small-pox; and on opening it with a needle, the contents were a ftraw-coloured opaque fluid. The inflamed fpace furrounding it was very narrow in proportion. The puftules on the body were conical, rather obtufe at the end; fome contained pus at the extremity, and others others were covered with a yellowifh-brown fcab. On the eighteenth, the child was playing out of doors.

Hence it is evident, that the puftules on the body of Job W. were variolous, probably derived from the fame fource as thofe of the patient in whom the difeafe broke out four days before him, and that the puftules on his arm were pure Cow-pox, both difeafes going on at the fame time; the lancet inferted into the puftule on the arm producing genuine Cow-pox in Elizabeth Titley, and that infected by the puftules on the knee, Small-pox in Hannah Titley. But I am inclined to believe, that the Cow-pox inoculation modified the progrefs of the Small-pox, which inftead of appearing on the face and bofom, broke out on the lower extremities, proceeding very gradually upwards.

I have feen the two difeafes exifting together in three other inftances. Mary Waffe, of Aftwith, 5 years old, after having juft gone through the chicken-pox, was inoculated by her mother, a very intelligent woman, in both arms, with Cow-pox virus, taken by her hufband on a lancet, two or three days before, from a patient inoculated by a cabinet-maker about three miles diftant, on the 24th of July. On the 6th day, one of the punctures appeared inflamed, and ftill more fo on the 7th, in the evening of which day fhe complained of not being well. On the 8th fhe had no appetite, could not walk, and was feverifh in the afternoon; and on the 9th the Small-pox broke out in the face. During all this time the inoculated puftule proceeded regularly, and on the 11th appeared to be at the height. The mother, from whom I received the above account, which I wrote down on the fpot, and who had inoculated one of her children fome years before with Small-pox, defcribed the inoculated puftules as being circular, lower in the middle than at the edge, and as appearing to contain a fluid of a bluifh white. On the 14th day, the 6th day of the eruption of Small-pox, I firft faw the patient. There was a degree of rednefs in the fkin round the inoculated puftule, fuch as remains after Cow-pox, and the puftule now fecreted fome yellow pus at the margin, as Cow-pox puftules fometimes do. There were no Small-pox puftules within the limits of the faded areola. The Small-pox I was informed went through its ufual courfe, and fome weeks after I faw the child perfectly recovered. The Small-pox had prevailed in the village fome time, and I doubt not, this child had received the infection previous to the infertion of the Cow-pox virus.

W. Drabbles of Inmanfthorpe, a year and a half old, was inoculated by Mr. Walker, furgeon, of this town, with Cow-pox virus, on the 6th day preceding that on which the Small-pox broke out. On the 3d day of the Cow-pox inoculation, the punctures appeared inflamed. I saw the child on the 8th day. They were true Cow-pox pustules, but several Small-pox pustules had made their appearance within the limits of the areola; and on the 12th day, the 8th of the Small-pox fever, the child died.

Another child of the same family was inoculated with Cow-pox virus, on the 4th day preceding that on which the Small-pox broke out, and the day before the commencement of the variolous fever. On the 3d day the punctures appeared inflamed, and on the 8th there were two Cow-pox pustules, with one of Small-pox, at the distance of half an inch from them. Mr. Walker informed me, that on the 12th day, the 11th of the Small-pox fever, the pustules looked all alike, the Cow-pox pustules not being surrounded by an areola. This patient recovered.

Hannah Drabbles, 6 years old, was inoculated with Cow-pox, on the 7th day preceding that on which the Small-pox broke out. On the 8th day, one of the punctures for the first time appeared slightly inflamed, and Mr. Walker informed me, that on the 12th, the 8th of the Small-pox fever, they all appeared exactly similar to the Small-pox pustules. This child also recovered.

The next caution I have to mention is, that all inoculators should strenuously inculcate the necessity of every patient being inspected at such periods of the disease, as may enable them to pronounce whether the patient is rendered secure from the action of Small-pox. At Ilkeston near Nottingham, two children were inoculated with Cow-pox virus. Eight weeks after they caught the Small-pox, and one of them died. Being in the neighbourhood, I extended my ride to inquire into the circumstances. Mr. Williams, the surgeon who inoculated them, carried me to the parents, from whom I learnt that the pustules rose in the course of a few days into elevated pustules, like those of distinct Small-pox, were never flat, were soon changed into a scab, and that the surrounding skin was not inflamed to more than half an inch from the base. I drew representations of convex and depressed pustules, as viewed in profile. The mother said they were like the convex, but never like the depressed. Hence it was evident, that the disease which the Cow-pox virus excited, was the crustaceous pustule described by Dr. Jenner,* and which Dr. Woodville, in his excellent Observations on the Cow-pox, has proved to afford

* Continuation of Facts and Observations relating to the Variolæ Vaccinæ, 1800, p. 35.

afford no fecurity againft the Small-pox. The gratification I felt at having difcovered the caufe of the failure could only be equalled by the furprife I experienced, at finding the new inoculation in high reputation in the village. They were all inoculated with Cow-pox. I earneftly recommended a re-inoculation of all the cafes which had not been regularly infpected, and whofe appearances had not been diftinctly recollected, left the popular enthufiafm in favour of the new inoculation fhould be damped by future failures.

The difcovery of the cruftaceous Cow-pox, (or *vacciola leprosa*, as I propofe to our Nofologifts to have it ftyled, diftinguifhing the prefervative againft Small-pox by the appellation of *vacciola scatellata*,) will enable us to reconcile the contradictory accounts of the effects of the cafual Cow-pox in fecuring the conftitution againft Small-pox, as related by Drs. Jenner and Pearfon, and the cafes publifhed in oppofition by Drs. Ingenhoufz and Beddoes. It is natural to fuppofe that, in fome inftances, the ulcers on the hands and arms affumed the cruftaceous form; and the contradictory evidence afforded by tradition, probably prevented the earlier introduction of the practice of Cow-pox inoculation.

Whenever any patient is reported to have the Small-pox after inoculation with Cow-pox virus, the inoculator fhould take the earlieft opportunity of infpecting the cafe. J. Burton, who had the Cow-pox in July, had the chicken-pox about a month ago. I am,

Dear Sir,

Your's, &c.

*Chefterfield, Nov.* 17, 1800. JONATHAN STOKES.

